# Retrospective Evaluation of Progenitor Biological Bandage Use: A Complementary and Safe Therapeutic Management Option for Prevention of Hypertrophic Scarring in Pediatric Burn Care

**DOI:** 10.3390/ph14030201

**Published:** 2021-02-28

**Authors:** Karim Al-Dourobi, Alexis Laurent, Lina Deghayli, Marjorie Flahaut, Philippe Abdel-Sayed, Corinne Scaletta, Murielle Michetti, Laurent Waselle, Jeanne-Pascale Simon, Oumama El Ezzi, Wassim Raffoul, Lee Ann Applegate, Nathalie Hirt-Burri, Anthony S de Buys Roessingh

**Affiliations:** 1 Plastic, Reconstructive and Hand Surgery Service, Lausanne University Hospital, University of Lausanne, CH-1011 Lausanne, Switzerland; Karim.Al-Dourobi@chuv.ch (K.A.-D.); alexis.laurent@unil.ch (A.L.); lina.m.deghayli@gmail.com (L.D.); Marjorie.Flahaut@chuv.ch (M.F.); Philippe.Abdel-Sayed@chuv.ch (P.A.-S.); Corinne.Scaletta@chuv.ch (C.S.); Murielle.Michetti@chuv.ch (M.M.); Wassim.Raffoul@chuv.ch (W.R.); Lee.Laurent-Applegate@chuv.ch (L.A.A.); Nathalie.Burri@chuv.ch (N.H.-B.); 2 Lausanne Burn Center, Lausanne University Hospital, University of Lausanne, CH-1011 Lausanne, Switzerland; Oumama.El-Ezzi@chuv.ch; 3 Regenerative Therapy Unit, Lausanne University Hospital, University of Lausanne, CH-1066 Epalinges, Switzerland; 4 Cell Production Center, Lausanne University Hospital, University of Lausanne, CH-1066 Epalinges, Switzerland; Laurent.Waselle@chuv.ch; 5 Unit of Legal Affairs, Lausanne University Hospital, University of Lausanne, CH-1011 Lausanne, Switzerland; Jeanne-Pascale.Simon@chuv.ch; 6 Children and Adolescent Surgery Service, Lausanne University Hospital, University of Lausanne, CH-1011 Lausanne, Switzerland; 7 Oxford Suzhou Center for Advanced Research, Science and Technology Co., Ltd., Oxford University, Suzhou 215000, China; 8 Center for Applied Biotechnology and Molecular Medicine, University of Zurich, CH-8057 Zurich, Switzerland

**Keywords:** fetal progenitor fibroblasts, pediatric second-degree burns, optimized cell banking, progenitor biological bandages

## Abstract

Progenitor Biological Bandages (PBB) have been continuously applied clinically in the Lausanne Burn Center for over two decades. Vast translational experience and hindsight have been gathered, specifically for cutaneous healing promotion of donor-site grafts and second-degree pediatric burns. PBBs constitute combined Advanced Therapy Medicinal Products, containing viable cultured allogeneic fetal dermal progenitor fibroblasts. Such constructs may partly favor repair and regeneration of functional cutaneous tissues by releasing cytokines and growth factors, potentially negating the need for subsequent skin grafting, while reducing the formation of hypertrophic scar tissues. This retrospective case-control study (2010–2018) of pediatric second-degree burn patients comprehensively compared two initial wound treatment options (i.e., PBBs versus Aquacel^®^ Ag, applied during ten to twelve days post-trauma). Results confirmed clinical safety of PBBs with regard to morbidity, mortality, and overall complications. No difference was detected between groups for length of hospitalization or initial relative burn surface decreasing rates. Nevertheless, a trend was observed in younger patients treated with PBBs, requiring fewer corrective interventions or subsequent skin grafting. Importantly, significant improvements were observed in the PBB group regarding hypertrophic scarring (i.e., reduced number of scar complications and related corrective interventions). Such results establish evidence of clinical benefits yielded by the Swiss fetal progenitor cell transplantation program and favor further implementation of specific cell therapies in highly specialized regenerative medicine.

## 1. Introduction

**Figure 1:**
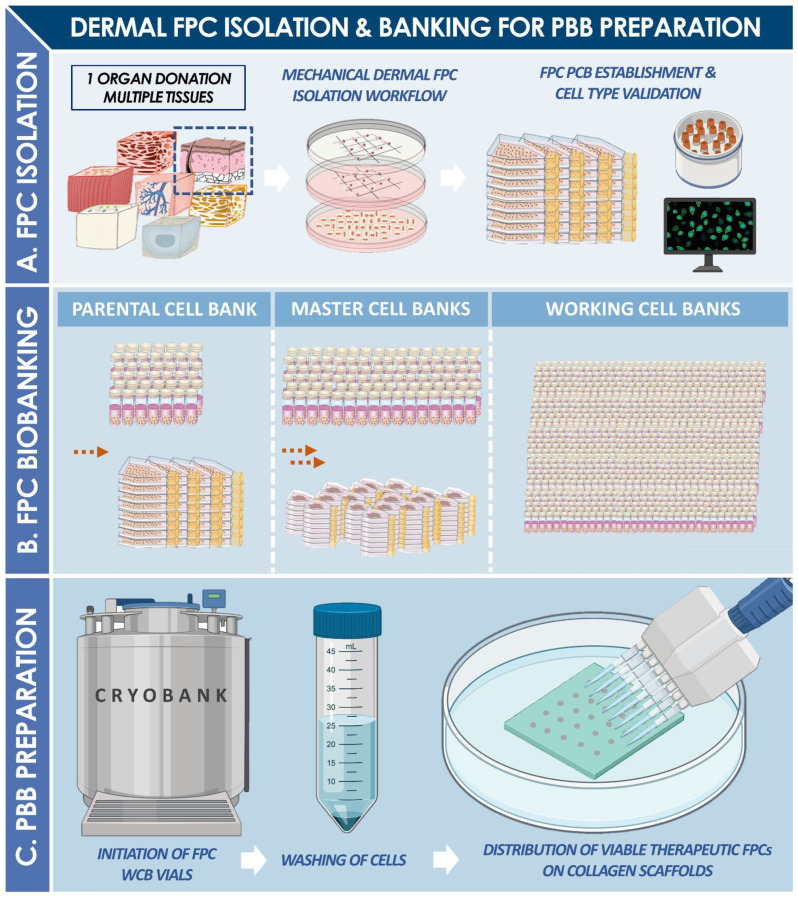
(**A**) Following a controlled fetal organ donation, multiple tissues may be traceably provided for processing, isolation, and establishment of primary fetal progenitor cell (FPC) types. After mechanical or enzymatic cell isolation and adherent in vitro culture initiation, parallel parental cell banks (PCB) may be established and used for preliminary cell type characterization. (**B**) Following defined technical specifications and within current good manufacturing practice (cGMP) workflows, extensive multi-tiered FPC cryopreserved progeny banks (i.e., master and working cell banks) are rapidly and consistently established, for efficient and sustainable exploitation of the cell sources of interest (e.g., dermal fibroblasts). (**C**) Upon request by the Burn Center physicians, the manufacture of Progenitor Biological Bandage (PBB) lots may be started on-demand, with initiation of allogeneic FPC vials at appropriate passages, washing of cells, and off-the-freezer distribution thereof on collagen scaffolds. After appropriate incubation, PBB lots are thereafter ready for safe release and transport to the operating theatre.

Vast translational clinical experience and hindsight have been gathered around the therapeutic use of Progenitor Biological Bandages (PBB) in the Lausanne University Hospital Burn Center over the past two decades [1,2,3,4,5,6]. Pediatric burn patient populations have constituted the primary beneficiaries of this form of cultured allogeneic fetal progenitor cell (FPC) therapy, initially implemented as a temporary coverage solution for skin autograft donor-sites and second-degree thermal cutaneous wounds [1,2]. Subsequently applied in diverse arrays of patients and applications (e.g., all degrees of burns except the first degree, graft donor-sites, sharp force trauma wounds, refractory chronic ulcers), PBBs have been locally coined as original “Swiss tools” for optimized management, reconstruction, and healing promotion of diversified cutaneous injuries and affections [7,8,9,10]. PBBs are currently classified as combined Advanced Therapy Medicinal Products (cATMP) or standardized transplants, as they are constituted by collagen sheet-scaffolds yielding cultured primary fetal dermal progenitor fibroblasts. Such bioengineered constructs may partly favor repair and regeneration of functional cutaneous tissues in burn victims by releasing cytokines and growth factors, potentially negating the need for subsequent skin grafting, while reducing the formation of hypertrophic scar tissues [4,6,9]. Inherently constituting a singularity, the Swiss FPC transplantation program, enabling the inception and continued clinical supply of PBBs, was devised for a holistic and sustainable control of therapeutic material generation and manufacturing under current Good Manufacturing Practices (cGMP) [11,12,13]. Therein, optimization and standardization of the successive steps necessary for implementation of FPC transplantation were undertaken, ranging from original primary FPC type establishment from a single organ donation, multi-tiered cell banking, to formulation of therapeutic biologicals for clinical delivery (Figure 1) [7,12,14]. This comprehensive quality-driven approach has enabled continuous optimization and challenging of the forefront of regenerative medicine in many musculoskeletal, cutaneous, and related applications, among which the management of burns constitutes a flagship to this day [13].

The development of PBBs was prompted by the high clinical necessity for effective therapeutic solutions in burn wound management, and was made possible by a biotechnology-driven approach of optimized sustainable cell sourcing. Indeed, severe cutaneous wounds such as burn trauma wounds are specifically characterized by complex healing dynamics and represent current unmet needs in clinical settings around the globe, especially in pediatric populations [15,16,17]. Standard surgical treatments and techniques are relatively limited for effective restoration of cutaneous structure and functionality [18]. Furthermore, accidental burns (e.g., thermal, chemical, electrical) are most common in pediatric populations and often occur in domestic environments [15,19]. In the specific case of second-degree burns, autologous skin grafting is often necessary at some point, to avoid lengthy or incomplete healing and hypertrophic scarring. Indeed, spontaneous cutaneous tissue repair or regeneration is generally poorly predictable and highly dependent on innate properties of the attained tissue itself (e.g., quality, age, location) [20]. Therein, indication for skin grafting is based on clinical examination by trained surgeons of the progression of spontaneous healing during the initial treatment period of ten to twelve days, during which patients are showered every two to three days to clean wounds and remove granulation tissue [18,21]. When a skin graft proves necessary, the gold standard consists in autologous tissue grafting (i.e., split-thickness autograft or Thiersch/Reverdin pinch graft), which may be supplemented with cultured epithelial autografts (CEA) or cultured dermal-epidermal autografts (CDEA) [22,23,24,25,26,27]. However, due to specific and inherent tech-nical issues in autologous cell-based therapeutic protocols (e.g., delays in product availability due to extensive culture periods), cell source selection was reoriented toward pragmatic valorization of FPC sources [28,29]. Primary FPC types are pre-terminally differentiated, with high expansion and regeneration potentials, and low immunogenic properties [30,31]. Technical simplicity of culture, potential for extensive cell banking, and robustness of primary FPC types have allowed for optimal adaptation thereof to in-house cGMP manufacturing workflows, creating a scalable, sustainable, and effective therapeutic material supply chain (Table 1). Indeed, a relatively short manufacturing delay by the pharmacy service of 18–24 h is assorted to PBBs, after prescription and ordering by the attending clinician [7,32]. Importantly, practical devising of serial and tiered cell banking workflows allows for the potential derivation of over 39 billion PBB therapeutic product units from the original bioprocessing of a single fetal organ donation. Therein, direct off-the-freezer seeding of viable fetal progenitor dermal fibroblasts on equine collagen sheet-scaffolds allows for on-demand serial and batch preparation of moldable single-use PBB wound coverages, which potentially mediate scarless wound healing [6,33,34,35,36,37]. 

Despite over twenty years of clinical experience and proven long-term safety with the primary or adjuvant use of PBBs, specific Swiss legal and regulatory requirements have prompted thorough evaluation of such regenerative medicine protocols or products, in view of determining or confirming safety and efficacy, using stringent standardized criteria [2,6]. Current prospective investigation of PBB safety and performance has been undertaken in Lausanne and has received ethical approvals, within a marketing authorization process, to establish formal risk-benefit ratios and to clinically validate protocols based on appropriate objective endpoints. Notwithstanding, on-going clinical trials (i.e., Clinicaltrials.gov ID numbers NCT03624023 and NCT02737748) have thus far substantiated the safety of the same source of fetal progenitor fibroblasts (i.e., FE002-SK2 FPC type) in various patient populations and applications (e.g., lower limb ulcers and split-thickness graft donor-sites). Despite updated regulatory requirements and increasing administrative pressure, continued use of PBBs is currently maintained in the Lausanne Burn Center. Indeed, the accumulated experience of specialized clinicians has allowed characterization of the efficacy of PBBs as unequalled by commercially available preparations and products. Therein and most importantly, direct impacts on morbi/mortality (i.e., vital rescue of extreme and severe burn wounds) and patient quality of life (i.e., averting the need for autologous graft harvest or enabling of single-site serial harvest) were practically outlined and have justified compassionate use. 

Within inherent design limitations, the present retrospective clinical study is intended to analyze available data to evaluate the use of PBBs compared to a control standard treatment, specifically for comparative evaluation of effectiveness and safety in second-degree pediatric burn patients (i.e., initial wound management over ten to twelve days post-trauma). The overall aim therein was to determine if PBB treatments lead to different/better outcomes than standard initial burn wound treatments (e.g., Aquacel^®^ Ag). Therefore, short- and long-term benefits of systematic PBB applications between 2010 and 2018 were quantified and described, by comparing the outcomes of a “PBB group” with a comparable group of pediatric patients treated with hydrofiber dressings (i.e., Aquacel^®^ Ag, practically replaced by PBBs in 2017). Both treatment options were exchanged every two to three days during showering, if necessary, depending on the degree of cutaneous recovery [38]. Specific endpoints of analysis comprised the need for secondary surgical/anesthetic treatments, hospitalization period length, and occurrence of early complications (i.e., infection, immune reaction, need for blood transfusions) and/or late complications (i.e., scars and sequelae). Overall safety of PBB applications was described and confirmed herein, along with specific advantages for management of second-degree pediatric burn wounds (i.e., enhanced functional and qualitative healing, with reduced long-term hypertrophic scarring). This work further strengthens the rationale for therapeutic FPC use and preliminarily introduces a standard clinical workflow, to be further defined in upcoming prospective studies around PBB implementation in Burn Centers. 

## 2. Results

### 2.1. Pediatric Burn Patient Demographics and Burn-Related Data 

**Figure 2:**
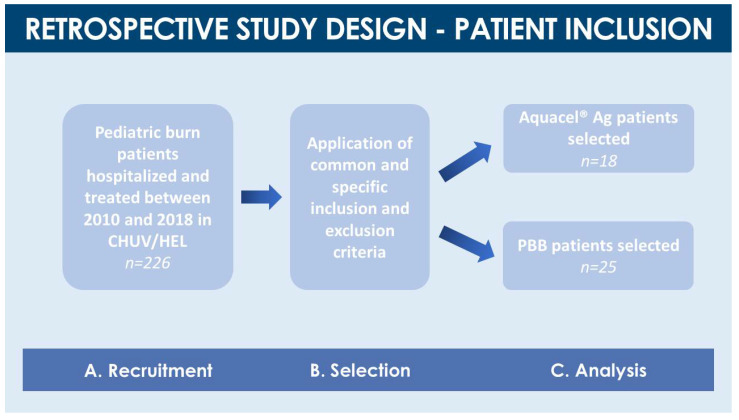
Flowchart of the studied pediatric burn patient population within the case-control retrospective study design. (**A**) A total of 43 patients was considered within two groups (i.e., Aquacel^®^ Ag group, *n* = 18, and PBB group, *n* = 25). (**B**) Common inclusion criteria comprised an age <18 years, attained Total Body Surface Area (TBSA) >10% for children >5 years, and attained TBSA >5% for children <2 years. Common exclusion criteria comprised depth or severity of burns not exceeding the first degree, and availability of documentation attesting to refusal of patient inclusion. (**C**) A specific exclusion criterion was applied, for the cases where PBBs were applied only on graft donor-sites and not on primary burn wounds. CHUV, Centre Hospitalier Universitaire Vaudois; HEL, Hôpital de l’Enfance de Lausanne; PBB, Progenitor Biological Bandage.

Among the 226 pediatric burn patient files considered, forty-three fulfilled our inclusion criteria (Figure 2). The age range of the total included patient population was comprised between 3.5 months and 15.5 years (Table 2). Half of the total patient population (i.e., 51.2%) had been referred to the Lausanne Burn Center by a third-party medical institution. 

**Figure 3:**
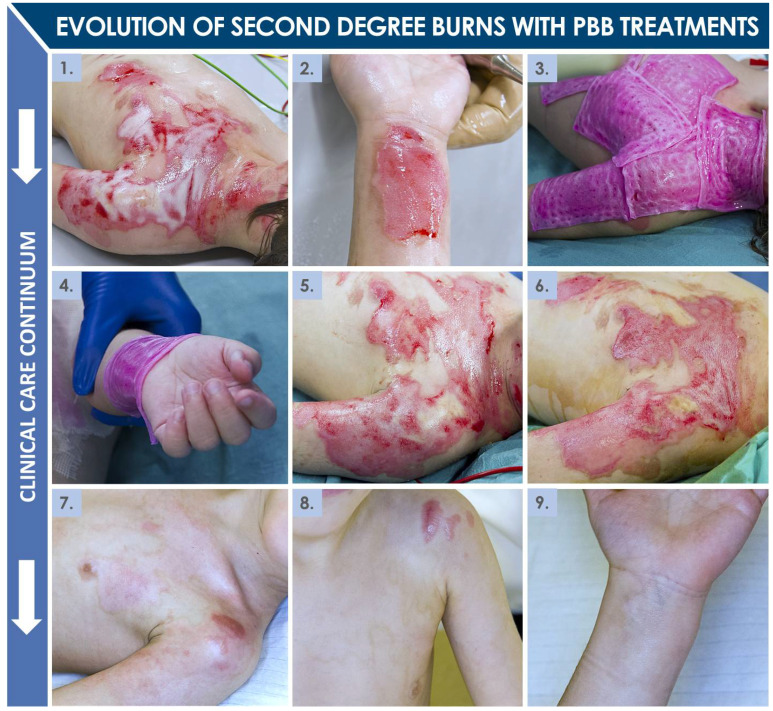
Second-degree deep scalding wounds of the upper body and limb (i.e., 12% TBSA) having been managed with PBB applications and without skin grafts. (**1**–**2**) Photographic imaging of the lesions after initial debridement and cleaning. (**3**–**4**) Initial PBB application on the wounds. (**5**–**6**) Photographic imaging of the upper body lesions after six and eight days of treatment, respectively. (**7**) Follow-up photographic imaging of the upper body lesions after six weeks of maintenance therapy (i.e., mechanical stimulation of wounds and pressure garment wear). (**8**–**9**) Follow-up photographic imaging of the lesions after fourteen weeks of maintenance therapy. PBB, Progenitor Biological Bandage. Modified and adapted with permission from Laurent et al. (2020) [13].

**Figure 4:**
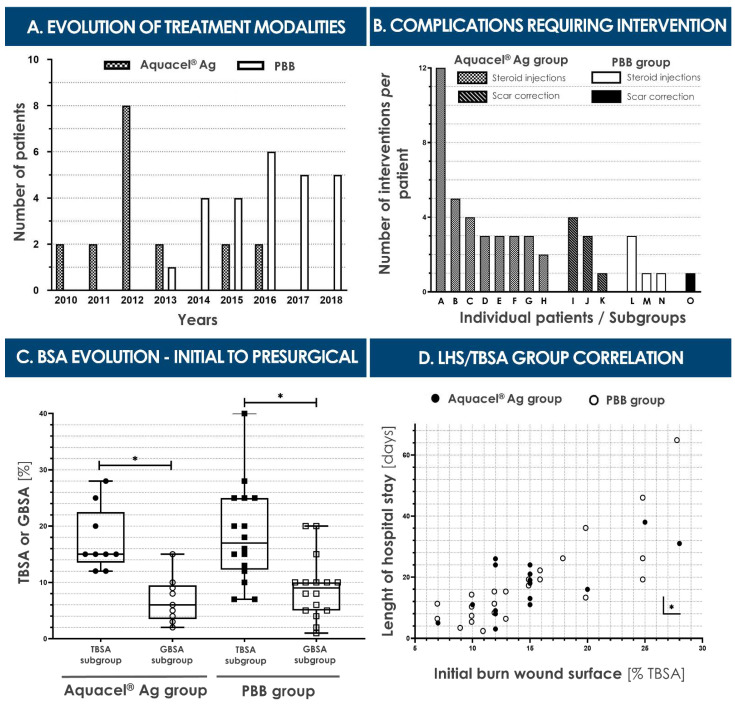
(**A**) Histogram presenting the evolution of initial burn wound treatment modalities (i.e., PBB or Aquacel^®^ Ag), for the patients included in the study. (**B**) Bar chart of interventions for delayed skin-related complications, representing individual patients on the X axis (i.e., in the different study and intervention groups, respectively) and the number of interventions in an outpatient setting on the Y axis. The probability of requiring steroid injections differed significantly between groups (i.e., *p* < 0.031, two-tailed Fisher’s exact test), showing an advantage of PBB use over Aquacel^®^ Ag for scar-related complications. (**C**) Boxplots representing the initial (i.e., TBSA) and presurgical (i.e., GBSA) body surface area percentages for both groups. Only the patients who benefited from grafting were considered. The following subgroups of data were compared using a Kruskal-Wallis test: (*i*) Aquacel^®^ Ag TBSA/GBSA, (*ii*) PBB TBSA/GBSA, (*iii*) Aquacel^®^ Ag TBSA/PBB TBSA, and (*iv*) Aquacel^®^ Ag GBSA/PBB GBSA. Results revealed partial statistical significance in the variance of medians (i.e., H = 21.92, approximative *p* < 0.001, two-tailed). Post hoc Dunn’s multiple comparison test showed statistical significance between Aquacel^®^ Ag TBSA/GBSA and PBB TBSA/GBSA, respectively (i.e., *p* < 0.004, two-tailed). No statistical significance in population distribution was shown between Aquacel^®^ Ag TBSA/PBB TBSA and Aquacel^®^ Ag GBSA/PBB GBSA (i.e., *p* > 0.999, two-tailed). (**D**) Spearman’s correlations between length of hospital stay (LHS) and affected TBSA for the included patient population (i.e., both groups). One patient from each group was excluded (i.e., child abuse or major social issues). Aquacel^®^ Ag group: *n* = 17, X axis median 15 ± 3% (7–28), Y axis median 16 ± 15 days (3–38), r = 0.63, *p* = 0.007, two-tailed. PBB group: *n =* 24, X axis median 13.0 ± 8.5% (7–28), Y axis median 14.5 ± 12.0 days (2–65), r = 0.83, *p* < 0.001, two-tailed. Asterisks were used to visually indicate statistically significative differences. GBSA, grafted body surface area; PBB, Progenitor Biological Bandages; TBSA, total body surface area.

In the cases of second-degree burn wounds included in the study, the mean percentages of affected total body surface areas (TBSA) were comparable between both patient groups, with a median of 15.0 ± 5.5% (7–40%) TBSA for the total included pediatric population (i.e., variability reported as interquartile ranges, IQR). The vast majority (i.e., 81.4%) of patients had suffered from accidental scalding water burns (Table 2, Figure 3). The most common areas of injury for the total included population were the anterior trunk and upper limbs (i.e., 79.1% and 69.8%, respectively, Table 2). Among patients included in the study, 18 were treated with Aquacel^®^ Ag dressings and 25 with PBBs during the initial period of ten to twelve days post-trauma (Figure 2). From 2010 to 2012, only Aquacel^®^ Ag dressings were used (i.e., 12 patients). Aquacel^®^ Ag dressings were also used in 2013, 2015, and 2016 on another six patients. PBBs were used from 2013 until the end of the time-period considered in this study (i.e., 2018). Furthermore, PBBs were the only treatments used in 2014, 2017, and 2018 (Figure 4A). Despite the restricted number of patients in both treatment groups, due to the relatively low incidence of pediatric burn wounds in Western Switzerland, both considered populations were considered as similar and comparable for the purpose of the present retrospective cohort comparison study (Table 2). 

### 2.2. Anesthesia Sessions for Burn Wound Care

The number of anesthesia sessions which had been required during acute burn wound care is reported in Table 3. General anesthesia for other purposes (e.g., upper airway endoscopic procedures) were excluded. General anesthesia for outpatient interventions were not considered either, because most of these operations were performed under nitrous oxide inhalation and/or local anesthetics. The median number of anesthesia was 5 ± 2 (2–12) in the Aquacel^®^ Ag group and 6 ± 2 (2–19) in the PBB group (Table 3).

### 2.3. Hospital Stay Specifics and Length of Hospital Stay 

The median length of hospital stay (LHS) was quite similar in value for both groups, with 16 ± 15 days (3–38) for the Aquacel^®^ Ag group and 14.5 ± 12 days (2–65) for the PBB group, respectively (Table 4). Overall, twenty-three out of forty-one (56.1%) patients spent time in the pediatric intensive care unit (PICU), with six children (35.3%) in the Aquacel^®^ Ag group and seventeen (70.8%) in the PBB group. For each group, the time spent in the PICU was, respectively, 9.0 ± 14.8 days (Aquacel^®^ Ag group; 2–27) and 6 ± 10 days (PBB group; 1 –25) (Table 4). One patient in the Aquacel^®^ Ag group was excluded from the study because of a prolonged hospitalization due to child abuse. A second patient, originally in the PBB group, was excluded because of a complicated familial situation which extended the hospital stay. There were no deaths in either patient group. As illustrated in Figure 4D, a statistically significant correlation between the attained TBSA proportion and the LHS was found in both groups.

### 2.4. Second-Degree Burn Wound Surgical Care

The depth and extent of the second-degree burn wounds were categorized as total (T), intermediate (I), and grafted (G) body surface area (BSA) (Table 3, Figure 4). These evaluations were made visually at the time of bandage application and exchanges by experienced surgeons. Data from all included patients were used to determine medians, value ranges, and IQRs (Table 3). For the Aquacel^®^ Ag group, the median TBSA value was 15 ± 3% (7–28%). For the PBB group, the median TBSA value was 13 ± 10% (7–40%) (Figure 4C). IBSA evaluations took into account the patients with certified second-degree burns which had required subsequent surgical intervention for effective wound coverage (i.e., second-degree deep burns requiring partial-thickness skin grafts) and the patients with intermediate burn wounds, for which the outcome was uncertain at the time of evaluation (i.e., meaning a skin graft was still avoidable by using standard Aquacel^®^ Ag or PBB coverages) (Table 3). Based on this consideration, six children were excluded from the Aquacel^®^ Ag group. The corresponding IBSA median value was therefore 9.0 ± 9.1% (1–20%) (Table 3). For the PBB group, three cases were excluded. The median IBSA value was then 7.5 ± 7.3% (1–38%) (Table 3). GBSA evaluations, representing the relative grafted surface *per* capita, concerned the grafted patient population (i.e., patients which required a skin graft after ten to twelve days). For the Aquacel^®^ Ag group, nine patients were grafted, with a median GBSA value of 6 ± 5% (2–15%). For the PBB group, sixteen patients were grafted, with a median GBSA value of 9 ± 5% (1–20%) (Table 3, Figure 4C). As presented in Figure 4C, significant decreases between initial (i.e., TBSA) and presurgical (i.e., GBSA) considered body surface areas in both groups were observed, but no significant differences were revealed when comparing respective TBSA or GBSA values between both groups.

### 2.5. Burn Patient Infectious Complications and Contaminations

#### 2.5.1. Cutaneous Infections and Contaminations

A total of four skin contaminations and/or infections had been reported among the forty-three patients included in the study, with infections declared in both considered patient groups (Table 5). In the Aquacel^®^ Ag group, one patient had presented a skin graft contamination by *Staphylococcus aureus*, with positive evolution under topical treatment and without the need for systemic antibiotic treatment (G1). Another patient of the same group had developed a *Pseudomonas aeruginosa* burn wound infection and had required oral medication (G2). In the PBB group, two wound contaminations had been reported (both G1). The following pathogens had been identified based on skin swabs performed on these patients: *Enterococcus faecalis*, *Proteus mirabilis*, *Bacillus cereus*, *Achromobacter xylosoxidans*, and *Enterobacter cloacae*.

#### 2.5.2. Urinary Tract Contaminations and Infections 

Thirteen urinary tract infections had been reported among the total included patient population (Table 5). Three cases out of four in the Aquacel^®^ Ag group had received specific oral treatments (all G2). The responsible pathogens had been identified as *Pseudomonas aeruginosa*, *Escherichia coli*, and *Enterococcus faecalis*. The fourth patient had presented a more severe infection by *Klebsiella pneumoniae* and *Proteus mirabilis*, and therefore had required parenteral treatment (G3). In the PBB group, four children out of nine had presented non-severe infections and had been appropriately treated orally (all G2). Except for one infection by *Enterobacter cloacae* and one fungal infection by *Candida albicans*, the bacteria involved were the same as those identified in the Aquacel^®^ Ag group. Five patients had been more severely infected, as two of them had shown systemic signs of infection and three of them had developed pyelonephritis. All of these patients had received appropriate intravenous medication (all G3). *Enterococcus faecalis* and *Escherichia coli* were the predominant pathogens identified in these five cases.

#### 2.5.3. Respiratory Tract Contaminations and Infections 

There had been thirteen cases of respiratory system infection, three in the Aquacel^®^ Ag group, and ten in the PBB group (Table 5). Of the three patients in the Aquacel^®^ Ag group, the first had presented bronchiolitis and had only required supportive care (G1). The second, burned by backfire, had developed spastic bronchitis with a secondary infection, and had been given an oral antibiotic (G2). The third had developed a severe pulmonary infection due to *Haemophilus influenzae*, *Streptococcus pyogenes*, and *Staphylococcus aureus*, which had been identified in a culture of his sputum. This last patient had been effe-ctively treated by parenteral medication (G3). In the PBB group, five patients had presented viral infections (i.e., *Adenovirus* and *Coronavirus* spp.) and were not given any specific treatment (G1). One of them had been identified to carry *Human metapneumovirus*, along with low concentrations of *Staphylococcus aureus* and *Haemophilus influenzae*, which had been judged to be commensal. In another two cases from the PBB group, bacterial cultures had enabled the identification of *Haemophilus influenzae* and *Moraxella catarrhalis*, and oral antibiotics had been administered (G2). Finally, three patients had needed parenteral antibiotics for pneumonia (G3), wherein *Streptococcus pneumoniae*, *Staphylococcus aureus*, and *Haemophilus influenzae* were the germs involved.

#### 2.5.4. Blood Contaminations and Infections 

In the Aquacel^®^ Ag group, one child had presented sepsis and a blood culture had revealed *Staphylococcus aureus* (Table 5). The patient had required parenteral support and antibiotics (G3). In the PBB group, one patient had developed sepsis following a pulmonary infection and *Streptococcus pneumoniae* had been isolated (Table 5). The patient had received appropriate intravenous antibiotics (G3). He had then developed bacteremia due to *Stenotrophomonas maltophilia* and *Achromobacter xylosoxidans* but had not required specific treatment. 

### 2.6. Various Complications during Burn Patient Hospitalization

#### 2.6.1. Pre-Shock and Shock Reactions 

In the PBB group, one patient (i.e., TBSA 15%) had presented a hypovolemic state and had required urgent care, although his situation was not categorized as immediately life-threatening (G3). Another child (i.e., TBSA 40%) in the PBB group had suffered a hemorrhagic shock (G4). With adequate perfusion, transfusion, and hemodynamic support, he had recovered without sequelae. No such cases had been reported in the Aquacel^®^ Ag group (Table 5).

#### 2.6.2. Need for Blood Transfusions 

Transfusion for anemia was systematically graded as G3. Five patients had benefited from it in the Aquacel^®^ Ag group, and six patients had benefited from it in the PBB group (Table 5). Of the five patients in the Aquacel^®^ Ag group which had received blood transfusions, four had required multiple transfusions. One child had developed critical anemia with hemoglobin levels at 5.2 g/dL. In the PBB group, three of the six children who had been transfused had required multiple transfusions. Two patients had presented anemia with hemoglobin levels below 6.0 g/dL.

#### 2.6.3. Catheter-Related Complications

Complications related to catheter use had been reported only in the PBB group and not in the Aquacel^®^ Ag group (Table 5). Two patients had received anticoagulation therapy following central venous thrombosis (i.e., internal jugular and femoral veins) (G3) and one patient had developed phlebitis on a peripheral access catheter and had required intravenous antibiotic treatment (G3). 

#### 2.6.4. Immune Reactions

One single case of immune reaction in the PBB group had been reported, which had required intravenous medication (G3) (Table 5). The patient, who was not known for previous allergic reactions, had presented a diffuse urticarial reaction four hours after the application of PBBs. No repercussions on cardiovascular, respiratory, or digestive systems had been noted. After appropriate intravenous antihistaminic therapy, the reaction had been considered as resolved. The patient had suffered a second untriggered episode of urticaria the following day, and an antihistaminic drug had been given again, with good response. No specific cause for the second reaction was found. Three days later, PBBs had been applied again and the patient had presented no observable reaction. Idiopathic versus drug-induced urticaria had been retained as differential diagnoses. No cases of immune reaction had been reported in the Aquacel^®^ Ag group (Table 5).

#### 2.6.5. Patient Social and Behavioral Issues

In the Aquacel^®^ Ag group, three patients were considered as presenting complications (Table 5). One infant had presented a depressive episode (G2) and two had presented difficulty eating (G2). One of these two patients had come from a problematic social environment, and further investigation had revealed a case of serious child abuse (G3). In the case of one patient in the PBB group, complex familial matters and lack of educational and parental care had led to a police and social enquiry. Child abuse had been excluded, but a specific and altered domestic framework had been set up after hospital discharge (G3) (Table 5). 

#### 2.6.6. Delayed Skin-Related Complications 

All delayed skin-related complications were classified as G2 (Table 5) and had been judged as requiring local, non-emergency intervention. The Aquacel^®^ Ag group had yielded eight cases of hypertrophic scars, and three patients had required Z-plasties or specific surgical intervention (i.e., lower limb, two on the abdomen, three on the neck). The PBB group had yielded three cases of hypertrophic scars, and one who had benefited from a Z-plasty (i.e., lower limb).

#### 2.6.7. Hypertrophic Scarring 

In the Aquacel^®^ Ag group, eight patients (i.e., 44.4% of the total group population) (Figure 2) had presented hypertrophic scars during follow-up and had required several topical corticosteroid injections (i.e., ten on upper limbs, thirteen on the neck, eight on the thorax, three on the abdomen, nine on the face and chin, and eleven on lower limbs) on an outpatient basis (Table 5, Figure 4B). The median number of injections was 3 ± 1 (2–12). One male patient, burned by backfire, had presented significant scars on his face and neck and had received a total of twelve injections and two laser treatments (result not shown). In the PBB group, three patients out of twenty-five (i.e., 12%) had received corticosteroid injections (i.e., five on lower limbs, two on upper limbs, two on the chin, and one on the flank), also on an outpatient basis, the median value being 1 ± 1 (1–3). The number of corticosteroid injections *per* patient differed significantly between the two groups (Table 5, Figure 4C).

#### 2.6.8. Surgical Scar Corrections 

In the Aquacel^®^ Ag group, three patients (i.e., 16.7% of the total group population) (Figure 2) had required Z-plasties. Two of them had needed a single intervention. The third, described earlier as the male individual burned by backfire, had required two Z-plasties and a full-thickness secondary skin graft (results not shown) on his neck, due to formation of a major scar. In the PBB group, one patient (i.e., 4% of the total group population) had required a single surgical repair intervention. These subsequent corrective surgical interventions had all been performed on an outpatient basis.

#### 2.6.9. Other Scarring Sequelae Management

One patient in the Aquacel^®^ Ag group had presented a case of trigger digit of the thumb and had been operated as an outpatient without complications (results not shown).

## 3. Discussion

### 3.1. Current Clinical Need for Effective Burn Wound Early Coverage Solutions

The high incidence of household accidents implicating young children and thermal sources such as hot water, irons, stoves, or open fires often results in severe cutaneous burn injuries necessitating swift, multidimensional, and effective therapeutic management [19]. Current yearly statistics on thermal wounds affecting pediatric patients remain elevated, despite high public health efforts allocated toward accident prevention and risk awareness. Early and appropriate wound coverage with standard specific bandages (e.g., DuoDERM^®^, Kaltostat^®^, Polymem^®^, Mepitel^®^ Ag, Aquacel^®^ Ag) and creams (e.g., Ialugen Plus^®^) is possible to a certain extent, but relatively large or deep wounds reaching into the dermis and underlying tissues necessitate advanced coverage solutions, to limit the long-term impact of traumatic cutaneous injuries on the lives of young children [39]. Recent research in tissue engineering and material science has been rapidly producing novel biomaterials and cellular substrates characterized by remarkable biological functions and application potential. Therein, many bioengineered solutions have been proposed (e.g., Allox^®^, Epicel^®^, OrCel^®^, ReCell^®^, Apligraf^®^, TransCyte™, Lyphoderm^®^) for burn wounds, with the objective of combining traditional surgical management with novel regenerative solutions, to optimally stimulate resurgence of normal tissue structure and function [40,41,42,43,44,45]. Despite high interest and considerable development efforts, commercialization rates of such complex biological products remain relatively low [46,47,48]. 

According to current classifications, extent or seriousness of burn wounds are categorized based on multifactorial observations and measurements, such as damage depth (i.e., most profound cutaneous histological layer attained) and relative body surface extent (i.e., TBSA) of the lesions. With relatively high occurrence of severe burns (i.e., >5–10% TBSA in children and >20% TBSA in adults, burns to the face, genitals, hands, feet, joints, and burns with inhalation or polytrauma), especially in pediatric populations, modern healthcare systems are required to propose appropriate robust highly specialized medical solutions [25]. Such approaches generally comprise a well-defined patient care continuum, ranging from pre-hospital patient transfer to long-term reeducation and follow-up (i.e., accompaniment by the pediatric surgeon, nursing staff, physiotherapists, and occupational therapists), yet clinical outcomes may optimally benefit from additional implementation of autologous and allogeneic cell therapies [18,49,50,51]. Therein, differential approaches may be highly useful for management of individual burn patients, which each represent complex multimodal clinical cases, as burn wounds are seldom homogenous in extent, depth, and related complications [50]. Inherent individual differences between patients further prompt the development of standardized treatment workflows, while favoring the use of personalized or versatile effective cell therapy products [20]. To this end, regenerative medicine protocols aim to optimally support the restoration and repair of damaged and destroyed tissues. Implementation of new therapies aiming to accelerate recovery from burn wounds and to improve the quality of subsequent scars is essential [16,17].

Regarding second-degree pediatric burn wounds, clinical care in the Lausanne Burn Center may be described as biphasic, with initial evaluation and maintenance treatment, potentially followed by autologous or allogeneic grafting based on clinician observations and treatment prescription. For second-degree burns, which are the most common in children (i.e., >five years of age or adolescents), the need for a skin graft is generally determined after ten to twelve days. During the initial treatment period (i.e., ten to twelve days post-trauma), pediatric burn patients are showered every two to three days, to clean the wounds and remove all necrotic and granulation tissue. Wounds may then be initially covered with a hydrofiber dressing (e.g., Aquacel^®^ Ag), which has the potential to absorb wound exudates and to favor granulation tissue formation. This hydrofiber dressing requires serial exchange procedures (i.e., at least two times) because of its eventual saturation with wound exudate. A direct alternative to such hydrofiber dressings for management of second-degree burn wounds has been the PBB, clinically implemented for the past twenty years in Lausanne for various cutaneous acute and chronic applications. The main objective of using such constructs is to stimulate the natural cutaneous healing process of the burn wound during the first ten to twelve days, and thus potentially avoid the need for an autograft or at least reduce the size of the grafted area [1,2]. Subsequently, in cases where autografts are required, PBBs may be further applied for promoting re-epithelialization of donor-site wounds. Furthermore, a secondary yet major objective of applying PBBs on burn wounds is the promotion of a qualitatively enhanced healing process, potentially scarlessly, with drastically improved esthetic and functional outcomes and reduced need for subsequent corrective interventions. Specifically considering this secondary objective, results of the present study confirm the overall high usefulness of PPB use in pediatric second-degree burn victims. 

### 3.2. Two Decades of Translational and Transpositional Experience around PBBs

As shown by the clinical work reported herein, as well as by previously published pre-clinical and clinical reports, extensive in-house experience and know-how have been accumulated in the Lausanne Burn Center, especially for the management of pediatric burn patient wounds and complicated geriatric ulcers [1,2,3,7,9]. Importantly, the quality of patient follow-up and the iterative research and development around novel cell therapies have been enabled by a continuity in the multidisciplinary team directly and indirectly involved in the patient care continuum. Vertical transmission of integrated clinical and bio-therapeutic knowledge and technical skill-sets is essential in order to guarantee perennity and continuity of optimal care provision. Furthermore, and in addition to the in-house maintenance of specialized know-how through continued translational efforts, multi-centric collaboration and dissemination of techniques in various countries has enhanced the development of the core biomedical technology supporting the use of PBBs [13,14]. Therein, comparison between collaborator groups of diversified protocols and various prototype cell therapy products at respective pre-clinical stages favors the development of sound strategies for assurance of therapeutic quality and regulatory compliance. 

Therein, considerable experience has been gathered in Switzerland around industrial transposition and upscaling of FPC-based therapeutic product manufacturing, enabling multiple technology transfers and multi-centric development efforts [6]. To date and most importantly, a single qualifying fetal organ donation (i.e., codename FE002, 2009) has yielded sufficient progeny biological materials for at least four clinical trials (i.e., including the results of the present retrospective study) between Switzerland and Asia, with ethics and regulatory approvals for such investigations from appropriate authorities (i.e., in Switzerland, Japan, Taiwan, and USA) [6]. Such endeavors were technically enabled by the extensive cell banking potentials and related yields of dermal fibroblast FPCs, as it has been established and validated that a robust parental cell bank (PCB) established from the previously mentioned organ donation could potentially yield over 39 billion individual 9 × 12 cm PBB units [6,9]. Specifically, in the context of elaboration of investigator’s brochures (IB) and investigational medicinal product dossiers (IMPD), extensive data (i.e., in vitro and GLP in vivo safety, composition, and putative mechanisms of action) were generated around the therapeutic cell source of interest (i.e., FE002-SK2 FPC type, deposited in 2012 in the ECACC, N°12070301-FE002-SK2). Such results and experience, combined with the robust design of the original Swiss FPC transplantation program, enable consolidated conclusions to be drawn about the FE002-SK2 cell source, with stringent cGMP manufacturing workflows and safe provision of high therapeutic value clinical care [13,29]. On-going clinical research around the cell source of interest shall further and synergistically contribute to the gathered experience around PBBs in Switzerland. Therein, through maintained and systematic efforts on clinical translation and implementation of standardized clinical protocols, both quality and efficiency of burn patient care shall eventually be optimized. 

### 3.3. Safe and Effective Clinical Use of PBBs in Pediatric Second-Degree Burns

The present retrospective cohort comparison study further demonstrates that PBB application on second-degree pediatric burns can be considered as safe and at least as effective as current initial standard treatments (i.e., Aquacel^®^ Ag) for early wound coverage up to twelve days post-trauma (Figure 4). Furthermore, considering short-term efficacy measures, PBBs had previously been shown in several instances to restrict, partially or totally, the need for subsequent skin autografting, and such benefits had been observed in several patients included in the present study (Figure 3) [1,2]. Most importantly, PBBs were found to generally be beneficial over the long term in reducing hypertrophic scarring in pediatric burn wound repair (i.e., reduced number of scar complications and related corrective treatments, Table 5, Figure 4). Such results are in line with the observations made in the clinic to date about the esthetic and functional gains enabled by the use of PBBs. Notably, the drastically (i.e., *p* = 0.031) reduced need for secondary corrective interventions (e.g., steroid injections for scar management) appears to be most significant in comparative analysis, yielding important quality of life gains for pediatric burn patients in particular (i.e., 11 patients with late-complications in the Aquacel^®^ Ag group versus four in the PBB group, Table 5, Figure 4). 

The clinical data presented herein for the purpose of standardized retrospective evaluation of PBB use was derived from the analysis of two relatively small and homogenous pediatric patient groups. The two treatment groups (i.e., Aquacel^®^ Ag and PBB) included 18 and 25 patients, respectively. No significant difference was noted between the two groups regarding the care pathway or the number of general anesthesia required during wound care. The initial median percentages of burned surfaces were also comparable for the two groups, with 15% and 13% of TBSA affected for patients treated with Aquacel^®^ Ag and PBBs, respectively (Table 3). Scalding water was the leading cause of burn trauma and wound creation (Table 2). A major difficulty in the evaluation of second-degree burns had been the depth, since it could generally only be clearly defined after ten to twelve days of treatment [20]. Initial evaluations of the TBSA had therefore been made at D_0_, followed by an intermediate evaluation (i.e., IBSA) of a combination of superficial-intermediate and deep second-degree burn wounds at D_5_, and a final confirmation of deep second-degree burns requiring surgery at D_10_ (i.e., GBSA) (Table 3). 

Importantly for treatment safety aspects, no infections of burn wounds had been observed in the PBB group (Table 5). The usual infections suffered by burned patients are urinary infections, respiratory infections, catheter infections due to the long-term use of a central catheter, or skin-mediated sepsis due to nosocomial contamination (i.e., frequent in >40% TBSA burns). Such complications had been observed across both treatment groups, without significant differences between groups, and shall be the focus of subsequent prospective investigations, for clear assessment of PBB effects on specific complication occurrence. In this perspective, and to further mitigate the risk of burn wound infection during treatment, the next generation of PBBs is currently being developed, coupling antimicrobial dendrimers with therapeutic FPCs, in order to achieve a dual effect of enhanced wound healing and controlled infection on large burn surfaces [6]. Furthermore, no confirmed immune rejection related to PBB application was noted in the PBB group. Interestingly, more patients with deep second-degree burns were found at D_5_ in the PBB group than in the hydrofiber group. However, hypertrophic scaring was eventually present in three patients of the PBB group and eight in the Aquacel^®^ Ag group, suggesting a major and important difference in healing evolution between the wounds of respective groups (Table 5). Overall, no statistical differences were found between groups regarding rates of wound healing, length of hospital stay, or occurrence of showering sessions and surgeries (Figure 4). Specifically, when individual requirements for hospital stays in the PICU were compared for the two patient groups, a statistical difference (i.e., *p* = 0.031) was found, wherein patients in the PBB group needed intensive care in more cases (Table 4). However, a large subgroup of PBB-treated patients (i.e., seven patients) was under two years of age, whereas the Aquacel^®^ Ag group contained no patients in this age range. Patient group sizes were quite small to draw any satisfactory conclusion about the length of hospital stay outcome, and it was not possible to prove that PBBs would reduce the period of hospitalization and global costs. Notwithstanding, a trend was observed in the PBB group, in which patients required relatively shorter stays in the PICU, despite the more frequent need for intensive care upon admission for this group (Table 4). Furthermore, although the wounds in the Aquacel^®^ Ag group were less severe in terms of depth and TBSA, late complications were more severe in terms of hypertrophic scarring and the need for subsequent surgical corrections (Table 5, Figure 4). This was the case, despite the fact that patient treatment pathways in both groups in terms of skin massage and use of pressure garment were identical, with the same two attending surgeons, nurses, physiotherapists, and occupational therapists. Based on such specificities, we consider that the stimulation of the natural repair of the skin very probably reduces the risk of developing hypertrophic scar tissues. Therefore, it is possible to conclude that major long-term functional, esthetic, and psychological benefits may be associated with the early use of PBBs versus standard care, as hypertrophic scarring may be both physically and psychologically debilitating, in particular for young children burned on extremities or on the head [2]. Further investigations of the long-term local effects of PBB use on hypertrophic scar formation after second-degree burn treatment shall enable more precise and quantifiable measurements of functionally relevant outcomes, using standardized methods [52,53,54,55,56,57,58].

### 3.4. Current Legal and Regulatory Limitations of PBB Clinical Use

Following specific shifts in the local legal and regulatory landmarks in 2007, the implementation and specific use of PBBs in a University Hospital context, for the treatment of its own patients, has been drastically modified in Switzerland [59]. Following clinical and industry best-practices, all new developments and eventual market-approvals of cell-based products and therapies are subject to stringent safety and quality standards, for optimal insurance of the provision of non-iatrogenic clinical interventions for the recipients. Disruptive changes in applicable European legislation have trickled down to Swiss hospitals, generating complex approval procedures with mitigated success rates, hindering the tangible development of many therapies and protocols, despite documentation of historic safe experience in many cases (e.g., autologous keratinocyte cultures implemented in Burn Centers since the 1980s) [4,59,60,61,62]. In particular, specific regulatory process complications or cost-related deadlocks have been restricting the capacities of public institutions to maintain clinical implementation of regenerative medicine products such as PBBs. Specifically, consideration of in vitro cell culture mitotic expansion as a substantial manipulation for the formulation of standardized transplants (i.e., in Swiss law) has oriented clinical applications and related product developments toward GMP requirements generally applied in classical pharmaceutical industries [63,64,65,66]. Therein, notwithstanding the increasing need for professional support regarding burdening regulatory submissions, the manufacturing costs of standardized transplants for University Hospitals have generally become prohibitive. Moreover, in some instances of retrospective evaluation of historically implemented and proven life-saving therapies, conflicts between national and supra-national regulatory guidelines have been detrimentally settled by local regulators. 

Public hospitals such as the CHUV in Lausanne have been prompted to develop varied strategies in view of adaptation to rapidly shifting regulations and requirements, to be able to maintain the quality of care required by their public health mission. Therefore, in addition to transition toward cGMP standards for in-house cell therapy and product manufacturing, great care has been allocated toward the classification and prescription context of novel cell-based therapies, to ensure compliance with overarching legal provisions while balancing inherent clinical and ethical responsibilities of physicians [67,68]. Therein, with a mandatory approach by legal exposure mitigation, several pathways have been used or contemplated, comprising hospital exemptions for institutions treating their own patients with unapproved protocols, compassionate use or exceptional authorizations in case of absence of alternative treatments, orphan drug pathways, or classification of treatments as magistral or officinal preparations [59,69,70,71,72,73]. Continued and iterative translational development efforts directed at implementation of safe and proven cell therapies in hospital clinical contexts shall enable eventual remolding of currently overly restrictive and unharmonized regulatory frameworks, sensibly benefiting to the optimized health betterment of numerous patients worldwide.

### 3.5. Further Standardized Clinical Evaluation of PBB Applications in Burn Care

The main limitations of the present study were its retrospective nature and the relatively small patient group sizes, due to the relatively low incidence of severe and/or extensive burns in pediatric populations. Nevertheless, methodical application of strict inclusion and exclusion criteria was followed in order to reduce selection bias. An inevitable practical issue, due to the development of the inflammatory response to tissue damage, consisted in the subjective assessment of attained TBSA percentages at the beginning of treatment, with constant wound evolution during the ten to twelve first days after the injury. Notwithstanding and importantly, results of this retrospective study confirmed historic observations made on PBBs and underlined that the use of specific banked progenitor cells (i.e., dermal FPCs) for second-degree burn wound management was perfectly safe and was associated with fewer subsequent cutaneous complications (e.g., hypertrophic scarring) and less frequent need for re-interventions (Table 5, Figure 4) [1,2,3,4,5,6].

Recent ethical validation has been granted in Lausanne by the Cantonal Commission of Ethics for Research in Human Subjects for an upcoming clinical trial on standard use of PBBs (i.e., standardized transplants under Swiss law) in the CHUV Burn Center. This phase 1/2 interventional, prospective, and randomized monocentric study (i.e., “Evaluation of the safety and effectiveness of PBBs in burn care”, codename *Bru_PBB*, CER-VD, BASEC-ID 2020-01873, 2020) will aim, over the next five to ten years, to include at least 76 burn patients, and to comprehensively study the therapeutic effects of PBBs for promotion of spontaneous wound healing in two study arms (i.e., application of PBBs on second-degree burns and donor-site wounds). With the overall goal of routinely implementing PBB treatments in the Burn Center with appropriate authorizations, objectives of this new study will comprise demonstration of efficacy (i.e., short-term and long-term) of PBBs for treating second-degree burns and donor-site wounds, as well as confirmation of the safety of PBBs. In particular, specific and standardized outcome measurements will be performed in order to allow enhanced analysis and quantification of local PBB effects, using assessments such as re-epithelialization rates, scar appearance and color, skin elasticity, viscoelasticity, long-term extension/retraction potential, and pliability [52,53,54,55,56,57,58]. More broadly, observational data will complete the aforementioned specific measures, comprising LHS, numbers of applied PBBs, numbers of required autografts and grafted areas, scar treatment modes and duration, and incidence of wound infections or adverse effects.

Notwithstanding the importance of the patient-centered clinical work, further developmental efforts shall be allocated to the continued optimization of quality of cellular therapies available to burn victims in Switzerland. By using PBBs, we were able to show that the use of specific cell-banked FPCs for burn victims is safe and related to fewer later complications, for instance hypertrophic scarring. FPCs have been previously shown to be stable, consistent, and safe, presenting a therapeutic potential of utmost interest [1,8]. Holistic optimization of novel cell therapies is therefore necessary for the sound development of products and protocols eventually enabling enhanced clinical success. Further pre-clinical and clinical investigations around the use of cultured FPCs therefore bare the potential of bringing safe, sustainable, and effective novel regenerative medicine solutions to the bedside of patients, as exemplified by the past two decades of experience with the use of PBBs. 

## 4. Materials and Methods

### 4.1. Retrospective Cohort Comparison Study Design and Pediatric Burn Patient Inclusion

The present retrospective cohort comparison study covered a period from January 2010 to December 2018 at the Children and Adolescent Surgery Service and the Burn Center of the Lausanne University Hospital (CHUV, Switzerland). The present study was approved by the Cantonal Ethics Committee (i.e., CER-VD, BASEC-ID 2017-01796, 2017). Early and late primary outcomes for the presented retrospective study comprised closure kinetics of burn wounds depending on the surface and severity thereof, need for skin grafting and surface of grafted wounds, number of PBB applications, treatment-related complications, complications unrelated to treatment, length of hospital stay (i.e., including in intensive care), need for scar management and modalities/duration thereof. Based on the primary outcome observations and data, the retrospective cohort comparison was undertaken, to compare the different types of burn wound coverages (i.e., Aquacel^®^ Ag versus PBBs) in pediatric burn victims. Criteria for inclusion in the study were the age of the patients (i.e., <18 years old), and burn wound characteristics, namely second- and third-degree burns affecting 10% or more of the TBSA (i.e., 5% for children younger than two years old). Criteria for exclusion from the study were an age of 18 years or more, less than 10% of TBSA burns, first-degree burns, evidence of third-degree burns at the time of injury, and availability of any documentation attesting to a refusal of patient inclusion. Selected patients were divided into two groups, depending on the initial wound coverage treatment they had received for second-degree burns (i.e., Aquacel^®^ Ag or PBBs, Figure 2). Both groups were retrospectively examined, with specific attention being paid to patient primary and secondary outcomes in terms of subsequent need for surgical/esthetic treatments and hospitalization time, in terms of early complications (i.e., infections, blood transfusions, immune reactions) and in terms of late complications (i.e., scarring and sequelae, need for corrective interventions). 

### 4.2. Description of Burn Wound Treatment Applications (Aquacel^®^ Ag and PBBs)

**Figure 5:**
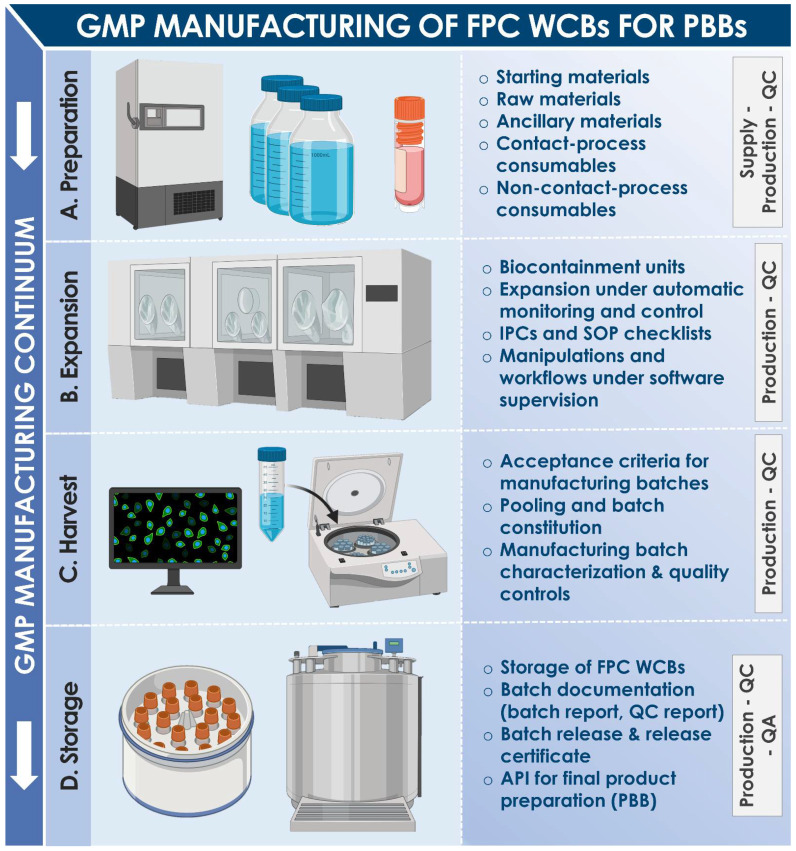
(**A**) All the required equipment, materials, and consumables necessary for the banking procedures are readied. (**B**) The in vitro mitotic cell propagation is performed in optimally inducive conditions under cGMP requirements. (**C**) Cellular material harvests are controlled for quality and used to constitute the production batches (i.e., cell bank lots). (**D**) Cell bank lots are cryopreserved until further expansion or use as active pharmaceutical ingredients in PBB manufacture. Post-production analysis of batch documentation allows for confirmation of acceptance and batch liberation, or rejection and batch discard. FPC, fetal progenitor cells; IPC, in-process control; PBB, Progenitor Biological Bandage; QA, quality assurance; QC, quality control; SOP, standard operating procedure; WCB, working cell bank.

**Figure 6:**
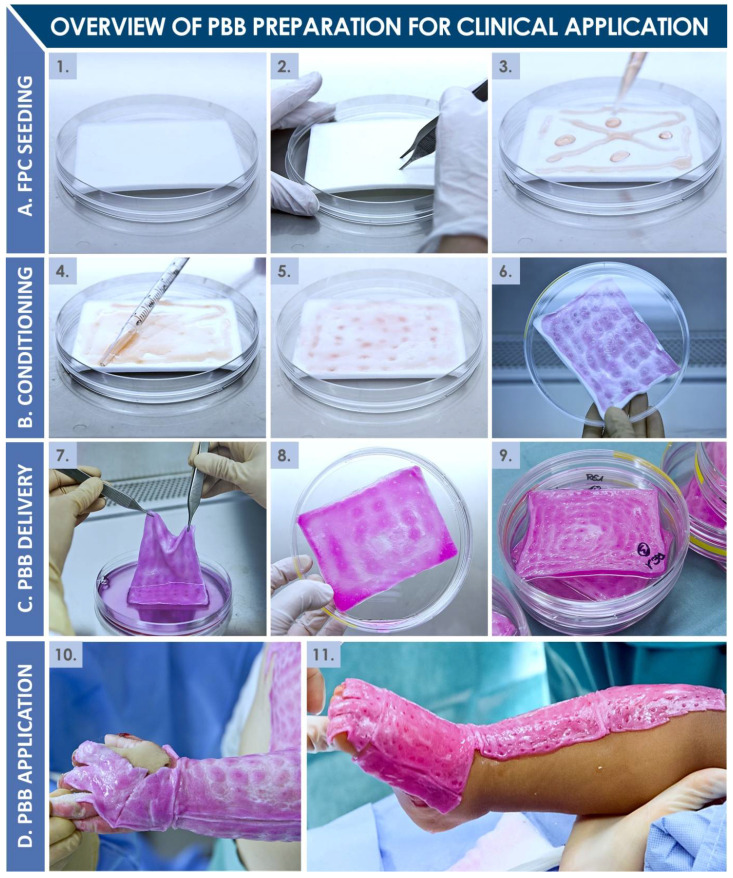
Photographic workflow of PBB manufacture and clinical application. (**A**) Equine collagen sheet-scaffolds are pre-conditioned by physical surface disruption and FPC suspensions are distributed thereon. (**B**) The cell suspension is homogenously distributed over the scaffold surface and allowed to be absorbed entirely, before cell growth medium is added to the vessel for construct incubation (i.e., 24–48 h at 37 °C under 5% CO_2_ in a humidified atmosphere). (**C**) PBBs are prepared for clinical delivery by rinsing and conditioning in individual vessels. Product quality controls are performed. (**D**) PBBs are applied as necessary after standard wound care, without staples or glue, on virtually all surfaces, before being overlayed with gauze and standard bandages. FPC, fetal progenitor cell; PBB, Progenitor Biological Bandage. Modified and adapted with permission from Laurent et al. (2020) [13].

PBBs (ref. PBF, CHUV Pharmacy Service, Switzerland) had been manufactured at the surgeon’s request by the in-house accredited Cell Production Center under cGMP standards and had been delivered directly to the operating room after a production period of at least 18 h but at the most 72 h following the start of production (Figure 5 and Figure 6). PBBs are composed of human dermal progenitor fibroblasts seeded on a biodegradable equine collagen scaffold (9 × 12 cm, KOLLAGEN resorb™, Resorba^®^ Medical GmbH, Germany, 4.5 × 10^3^ viable cells/cm^2^). Cellular materials had originated from a single fetal organ donation under the Swiss FPC transplantation program, after adequate processing and preservation in a multi-tiered biobanking workflow (Figure 1 and Figure 5). Once produced, PBBs are maintained in an appropriate transport medium in controlled conditions. Before transfer to the operating room, the constructs are rinsed with a saline buffer solution (ref. FE2010109, Bichsel AG, Unterseen, Switzerland) and conditioned in sealed plastic bags. The detailed techniques of FPC type isolation and establishment, tiered cell bank manufacturing (i.e., parental, master, and working cell banks), PBB manufacturing, and ethical or legal considerations were widely described in the literature [3,6,7,8,9,10,11]. In the Burn Center, PBBs had been placed directly on the lesions and covered with vaseline-coated gauze (Jelonet^™^, ref. 7404, Smith & Nephew S.A.S., Neuilly-sur-Seine, France). Regular bandages (compresses) had been used to protect the construct and the gauze. No stitches, staples, or biological glue had been needed [1,2]. Due to their composition, PBBs naturally degrade over the course of the two to three days between bandage exchanges, with residues being washed away during showering. On the other hand, the commercially available Aquacel^®^ Ag hydrofiber dressing (ref. 403708, ConvaTec, UK) retains excess wound exudate by vertical absorption, allowing fibrin to accumulate between the dressing and the wound.

The state of the dressing is evaluated every 48 to 72 h after application, and the saturated part of the dressing is exchanged. With time, when the whole dressing becomes saturated with exudate, it detaches spontaneously [38]. Aquacel^®^ Ag hydrofiber dressings are applied in the same manner as PBBs and overlayed with standard gauze and bandages. 

### 4.3. Pediatric Burn Patient Clinical Care Pathway

**Figure 7:**
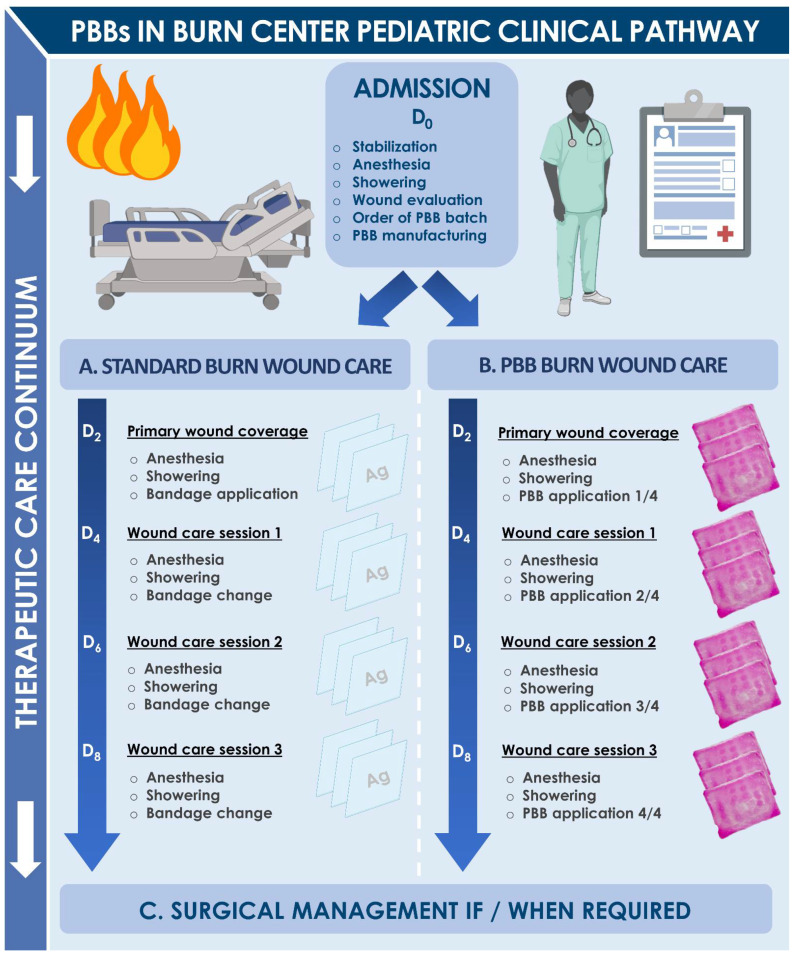
Schematic overview of the pediatric burn patient care workflow in the Lausanne Burn Center. After arrival at the emergency ward and stabilization, patients are brought to the Burn Unit. Under general anesthesia, patients are showered and scrubbed, and a first evaluation of the extent and depth of the burn wounds is made by the medical team. (**A**) Up to 2013, Aquacel^®^ Ag dressings or a healing and antiseptic cream such as Ialugen^®^ Plus were initially applied on second-degree burns. Every 48 to 72 h, Aquacel^®^ Ag dressings were evaluated, and exudate-saturated parts of the dressing were cut out before a new product application was performed, based on specific needs. (**B**) Since 2013, second-degree burns were preferably treated with PBBs instead of Aquacel^®^ Ag dressings. After evaluation of burn wounds, PBBs are regularly exchanged after 48 to 72 h under aseptic conditions in the operating room, and are applied on average three to four times to stimulate wound closure and tissue repair. (**C**) After an initial treatment period of ten to twelve days, the decision is made by clinicians to proceed with a skin graft or not, based on the observed evolution of the wounds and specific patient needs. PBB, Progenitor Biological Bandages.

Following an initial evaluation of each patient, including airway examination and fluid resuscitation, an estimate of both the percentage of affected TBSA and the depth of the burn wounds had been documented, and initial treatment had followed the usual care process for both groups, with a tetanus booster shot if needed and bacteriological examination by skin smear. The specific initial burn patient care pathway is summarized in Figure 7. Upon patient arrival, treatment was initiated under anesthesia with showering and irrigation using 0.9% sodium chloride (ref. 1000090, Bichsel AG, Switzerland), a *Matricaria chamomilla* extract at 2% dilution, and Ialugen^®^ Plus cream (ref. 45916, IBSA, Switzerland). All pediatric patients suffering from second-degree burns were medically evaluated by two senior surgeons specialized in the care of burned children. The following day, patients were put under anesthesia for a shower and thorough cleaning of burned tissue. Photographs had been taken then, and subsequently at each step of the treatment continuum. Thereafter, over the course of ten to twelve days, patients had received alternative wound coverage treatments (i.e., Aquacel^®^ Ag or PBBs) iteratively, provided that informed consent had been appropriately granted for the treatment with PBBs. Final appreciation of the depth of the burn wounds had been performed at days ten to twelve, when the decision had been made whether or not to perform an autologous skin graft. If, before days ten to twelve, the use of PBBs had led to appropriate wound epithelialization (i.e., >95% of wound surface), or if the hydrofiber dressing had adhered to the wound, grafts had not been deemed necessary. The surgeon’s evaluation of the wound healing process had determined the number of treatment procedures, ranging from three to four successive Aquacel^®^ Ag or PBB applications within a two-week period. If necessary, casts had been applied to maintain patient arms and feet in a neutral position. After appropriate closure of skin wounds, regular cream application had been initiated, and individually fitted pressure garments had been prepared for day and night use, for a period of four weeks to several months after trauma, depending on the evolution of the wounds and scars.

### 4.4. Specific and General Burn Patient Complications

Aquacel^®^ Ag and PBB safety were retrospectively evaluated in the course of patient treatment on the basis of the occurrence and type of complications, and by the analysis of photographs. All the relevant complications were graded using the Common Terminology Criteria for Adverse Events (CTCAE, v5.0, November 2017) on a scale of 1 (i.e., minimal intervention needed) to 5 (i.e., death). The number of infections in both groups were reported. Skin infections/contaminations on the site of the burn wound were recorded separately from those of other sites (i.e., blood, respiratory and urinary tracts). Complications were classified based on the need for specific treatment which they elicited. Simple antiseptic care or monitoring were graded as 1 (i.e., abbreviated G1 hereinafter). Oral antibiotic treatment or minimal impact were graded as G2. Parenteral antibiotic treatment or major impact were graded as G3. Life-threatening situations were graded as G4. Safety of considered wound coverages was also assessed according to specific literature regarding the origin, manufacture, and previous clinical use of PBBs [1,2,3,4,5,6,7,8,9,10,11,31,33]. 

### 4.5. Clinical Data Collection and Statistical Analysis

All the data included or excluded for the purpose of this retrospective study were collected from medical archives and acquired through the internal software at the CHUV (Siemens Soarian^®^, Archimede, and FileMaker) and reported in an Excel chart (Microsoft Corporation, USA). Patient names were coded by numbers. A password-protected file was created to link the different data to specific patients. All the data judged to be essential and relevant were reported in an Excel chart that was also password-protected. The physical and print documents were kept under lock and key. The extent of the burns (i.e., excluding sunburns or first-degree burns), was represented by the percentage of affected TBSA, expressed in median values with respective value ranges and interquartile ranges (IQR), as described in the operating protocols, and reported according to the Lund and Browder chart. The surgical BSA for second-degree burns had also been represented in median percentages, value ranges, and IQR. With respect to inclusion and exclusion criteria, collected parameters comprised patient and burn demographics, acute and delayed skin-related complications, general complications, affected body surface area, anesthesia frequencies, and hospital stay specifics.

The number of days of hospitalization was taken into account and expressed in median values of days with measures of spread by value ranges and IQRs. LHS included the time spent on the pediatric floor and, in some cases, the time spent in the pediatric intensive care unit (PICU). The complicated cases of child abuse or social issue were excluded from the analyses. Because of limited numbers of cases and study design, descriptive statistics and non-parametric tests were used. Quantitative data were expressed either in frequencies with percentages or in medians with measures of spread by value ranges and IQRs. Qualitative data were represented in frequencies with percentages. For clarity, percentages were rounded up to the nearest one decimal place and a minor deviation of 1% at most was deemed acceptable in additions. For categorial variables, Fisher’s exact test was employed. For continuous variables, Mann-Whitney U-test or Kruskal-Wallis tests were applied to assess independent group differences. In the case of multiple tests, post hoc Dunn’s multiple comparison test was performed. Correlation analysis was achieved by Spearman’s rank-order correlation. A *p*-value < 0.05, two-tailed, was considered statistically significant. The calculations were performed using Excel and GraphPad Prism v. 8.0.2 (GraphPad Software, Inc., San Diego, CA, USA).

## 5. Conclusions

Regulatory requirements in the field of tissue engineering are shifting and impact many historically implemented cell therapies in Burn Centers. Rigorous clinical studies are current high priorities for public hospitals, which seek to maintain the use of highly effective interventions. Over twenty years of experience of the use of PBBs and pioneering biomedical developments in the Lausanne Burn Center have generated robust hindsight and technical know-how for cell-based therapy translation and transposition. The present retrospective study results validate existing safety and efficacy data on PBB use in pediatric burn patient populations, underlining and confirming the safety of such treatments. Furthermore and most importantly, despite statistically comparable short-term observations, the long-term benefits of PBB applications (i.e., rather than Aquacel^®^ Ag dressings) were shown to be of high value, with observed reduction in hypertrophic scarring and reduced need for secondary corrective interventions. With enhanced quality of repaired tissues, and partially alleviated pain, the results presented herein speak in favor of continued implementation of PBBs as standards of care in the Burn Center. An upcoming standardized and prospective clinical trial on PBB use shall seek to further confirm effectiveness of such products, in order to ensure compliance with regulatory requirements. Overall, holistic comprehension has appeared as key in generating translational experience around regenerative therapies for treating pediatric burn patients. Continued endeavors toward quality optimization and standardized process development for PBBs and related FPCs in Switzerland and around the globe exemplify the tenacity and resolve necessary for attaining tangible therapeutic success. Such results highlight clinical benefits yielded by the Swiss FPC transplantation program and favor allocation of further efforts toward development and implementation of specific allogeneic cell therapies in highly specialized regenerative medicine.

## 6. Patents

Applegate, L.A. Preparation of parental cell bank from foetal tissue. 2013, *WIPO*, WO2013008174A1.

## Figures and Tables

**Table 1:** Overview of diverse characteristics of primary fetal progenitor cells (e.g., FE002-SK2 cell type) to be used as active pharmaceutical ingredients for the manufacture of regenerative medicine products such as Progenitor Biological Bandages (PBBs) or as biotechnological substrates [3,6,8,28,29].

Allogeneic Primary FPCs for Use as cATMP APIs	Technical Characteristics of the Dermal FE002-SK2 FPC Type	Proposed Functionality and Therapeutic Mechanisms of FPCs
Comprehensive traceability and safety screening of tissues	Single fetal organ donation	Intercellular contacts
Simple in vitro mitotic cell growth requirements	Extensive manufacturing lifespan (i.e., >12 in vitro passages)	Reversal of apoptotic signals or effects
High rate of mitotic proliferation	Excellent cell preservation in liquid nitrogen storage	Release of microvesicles and related secretome
Extensive and sustainable cell banking potential	Sustainable supply (i.e., >39 billion constructs available from one PCB)	Generation and deposition of extracellular matrix
High quality and safety of defined cell types	Stable pre-terminally differentiated phenotype	Homologous specific cellular functions
Robust biological material processing workflows	Stable karyotype	Paracrine and/or trophic modulation of endogenous cells
On-demand preparation of therapeutic products	Universal allogeneic donor material	Anti-inflammatory effects
Low immunogenicity and no tumorigenicity of APIs	Documented safety (i.e., in vitro, ex vivo, pre-clinical, and clinical)	Stimulation of cell proliferation, migration, and differentiation

API, active pharmaceutical ingredient; cATMP, combined advanced therapy medicinal product; FPC, fetal progenitor cell; PBB, Progenitor Biological Bandage; PCB, parental cell bank.

**Table 2:** Demographic variables and baseline characteristics of considered pediatric burn patients included in the case-control retrospective study, along with statistics on burn wound origin or mechanism and attained anatomical regions.

Category/Parameter	Male Patients	Female Patients	Total Population
	*N*^1^ = 22 (51.2%)	*N* = 21 (48.8%)	*N* = 43
**Age *n*^1^**			
0–2 years	12 (54.5%)	4 (19.0%)	16 (37.2%)
2–7 years	7 (31.8%)	15 (71.4%)	22 (51.2%)
7–12 years	1 (4.5%)	1 (4.8%)	2 (4.7%)
≥12 years	2 (9.1%)	1 (4.8%)	3 (7.0%)
**Sex ratio** (male/female)	NA	NA	1.05
**Admission *n*** **; primary/**	9 (40.9%)/13 (59.1%)	12 (57.1%)/9 (42.9%)	21 (48.8%)/22 (51.2%)
**secondary**			
**TBSA % (±IQR)**	14.0 ± 7.3 (7–40)	15 ± 5 (7–28)	15 ± 5.5 (7–40)
**Burn origin *n***			
Scalding (water)	19 (86.4%)	16 (76.2%)	35 (81.4%)
Scalding (oil)	1 (4.5%)	4 (19%)	5 (11.6%)
Flame	2 (9.1%)	1 (4.8%)	3 (7.0%)
**Location of lesions ^2^*n***			
Face	11 (50.0%)	9 (42.9%)	20 (46.5%)
Neck	10 (45.5%)	4 (19.2%)	14 (32.6%)
Upper limbs	17 (77.3%)	13 (61.9%)	30 (69.8%)
Lower limbs	10 (45.5%)	15 (71.2%)	25 (58.1%)
Anterior trunk	17 (77.3%)	17 (81.0%)	34 (79.1%)
Trunk dorsum	7 (31.8%)	2 (9.6%)	9 (20.9%)

^1^*N* and *n*, numbers in absolute values. ^2^ In the first group, 21 patients presented burns on multiple sites (i.e., five patients with two locations, four patients with three locations, 11 patients with four locations, and one patient with five locations). In the second group, 20 patients presented burns on multiple sites (i.e., nine patients with two locations, five patients with three locations, four patients with four locations, and two patients with five locations). IQR, interquartile range; NA, non-applicable; TBSA, total body surface area (i.e., first-degree burns excluded, expressed in median values, ranges, with interquartile range values).

**Table 3:** Evolutive burn wound BSA evaluations and number of general anesthesia sessions required for initial wound management according to treatment groups, with assorted statistical data.

Parameter	Relative Date and Degree of Wound Depth Assessment ^1^	Aquacel^®^ Ag Group *N* = 18		PBB Group *N* = 25		*p* Values
		*n* of Patients	Extent of Burns ^2^	*n* of Patients	Extent of Burns	
**TBSA %**	D_0_ (i.e., Initial OVE) *Second-degree superficial and deep burns*	18	15 ± 3 (7–28)	25	13 ± 10 (7–40)	*0.946*
**IBSA %**	D_5_ (i.e., Intermediate OVE) *Potential and certified second-degree deep burns*	12	9.0 ± 9.1 (1–20)	22	7.5 ± 7.3 (1–38)	*0.701*
**GBSA %**	D_10_ (i.e., Presurgical OVE) *Certified second-degree deep burns*	9	6 ± 5 (2–15)	16	9 ± 5 (1–20)	*0.331*
**Number of general anesthesia** **sessions *per* patient ^3^*n***		5 ± 2 (2–12)		6 ± 2 (2–19)		*0.173*

^1^ Certified second-degree deep burns at D_10_ were systematically grafted. ^2^ Body surface area percentages are expressed in medians, ranges, and interquartile ranges. Calculations were based on respective population distributions. ^3^ Numbers in absolute values expressed in medians, ranges, and IQR. BSA, body surface area; GBSA, grafted body surface area; IBSA, intermediate body surface area; TBSA, total body surface area; *N* and *n,* numbers in absolute values; OVE, operator’s visual evaluation; PBB, Progenitor Biological Bandages.

**Table 4:** Hospital stay data of included pediatric patients according to treatment groups, with mention of specific requirements for admission to pediatric intensive care ^1^.

Parameters	Aquacel^®^ Ag Group	PBB Group	*p* Values
	*N* = 17	*N* = 24	
**Admission *n*; primary/secondary**	7 (41.2%)/10 (58.8%)	13 (54.2%)/11 (45.8%)	*0.53*
**Length of hospital stay ^2^*d***	16 ± 15 (3–38)	14.5 ± 12 (2–65)	*0.728*
**Patients who needed PIC *n***	6/17 (35.3%)	17/24 (70.8%)	*0.031* *
0–2 years	NA	7/17	
2–7 years	5/6	9/17	
7–12 years	NA	NA	
≥12 years	1/6	1/17	
**Length of stay in PIC ^3^*d***	9.0 ± 14.8 (2–27)	6 ± 10 (1–25)	*0.572*

^1^ One patient was excluded from each group (i.e., cases of child abuse or major social issues). ^2^ Starting from hospitalization in the CHUV, including the days spent in the PIC unit. ^3^ Only the patients who benefited from PIC were considered. An asterisk was used to visually indicate statistically significative differences. *N* and *n,* numbers in absolute values; *d*, number of days, expressed in median values (range; interquartile range, IQR). CHUV, centre hospitalier universitaire Vaudois; NA, non-applicable; PIC, pediatric intensive care.

**Table 5:** Pediatric burn patient complications (i.e., specific and general) across both treatment groups during second-degree burn wound preliminary care (i.e., ten to twelve days post-trauma).

Types of Complications	Aquacel^®^ Ag Group	PBB Group	Complication Severity Grades According to the Current CTCAE	
	*N* = 18	*N* = 25	Aquacel^®^ Ag Group	PBB Group
**Acute Burn-Related Complications *n***				
Skin infections	1	0	1 × G2	NA
Sepsis	1	1	1 × G3	1 × G3
Pre-shock state/Shock	0	2	NA	1 × G3; 1 × G4
Social and behavioral issues	3	1	2 × G2; 1 × G3	1 × G3
**Delayed Burn-Related Complications *n***				
Hypertrophic scars	8	3	8 × G2	3 × G2
Surgical scar correction	3	1	4 × G2	1 × G2
Other scarring sequelae	1	0	1 × G2	NA
**Other Complications During**				
**Hospital Stay ^1^*n***				
Proven skin contaminations	1	2	1 × G1	2 × G1
Urinary tract infections	4	9	3 × G2; 1 × G3	4 × G2; 5 × G3
Respiratory tract infections	3	10	1 × G1; 1 × G2; 1 × G3	5 × G1; 2 × G2; 3 × G3
Catheter-related infections	0	3	NA	3 × G3
Transfusions for anemia	5	6	5 × G3	6 × G3
Drug hypersensitivity reactions	0	1	NA	1 × G3
Deaths	0	0	NA	NA

^1^ Ophthalmologic and metabolic complications were excluded. *N* and *n*, numbers in absolute. G1-5, grading of patient complications according to severity, ranging between 1 (i.e., minimal) to 5 (i.e., death). NA, non-applicable; PBB, Progenitor Biological Bandages; CTCAE, common terminology criteria for adverse events.
